# Iron overload and liver function in patients with beta thalassemia major: A cross sectional study

**DOI:** 10.12669/pjms.40.9.8961

**Published:** 2024-10

**Authors:** Amna Faruqi, Tooba Zafar, Sikander Subuctageen, Irfan Afzal Mughal

**Affiliations:** 1 Amna Faruqi, MBBS, MPhil, FCPS Department of Physiology, NUST School of Health Sciences, Islamabad, Pakistan; 2 Tooba Zafar, MBBS, FCPS Department of Physiology, NUST School of Health Sciences, Islamabad, Pakistan; 3 Sikander Subuctageen, MBBS Department of Ophthalmology, Fauji Foundation Hospital, Rawalpindi, Pakistan; 4 Irfan Afzal Mughal, MBBS, MPhil, PhD Department of Physiology, Akhtar Saeed Medical College, Lahore, Pakistan

**Keywords:** Hemoglobinopathies, Thalassemia, Iron overload, Liver function tests

## Abstract

**Objective:**

In thalassemia major, repeated blood transfusions result in iron overload causing organ damage. The objective of this study was assessment of liver enzymes in patients with Thalassemia major and to observe their association with ferritin.

**Method:**

A cross-sectional study was performed, at Islamabad Medical and Dental College and its affiliated Akbar Niazi Teaching Hospital from November 2021 till August 2022. Serum ferritin, AST, ALT, and total bilirubin levels were determined, in 135 patients of beta thalassemia major receiving transfusions. Data analysis was performed using SPSS Version 20. For categorical variables, calculation of frequencies and percentages was performed. Mean (± standard deviation) was determined for quantitative variables. ANOVA with post hoc Tukey’s test was used for determining association between liver enzymes and serum ferritin. A p-value of <0.05 was considered significant. The correlation between ferritin and LFTs was determined by Pearson’s correlation coefficient.

**Results:**

Patients had an age range of 7-30 years, and males constituted 51% of sample. Mean level of ferritin was 6062.61 + 3641.79 ng/ml, with an insignificant difference between the genders (*p* =0.366). The levels of AST, ALT and bilirubin were perceived to show a significant increase in patients with ferritin levels >5000ng/ml, when compared with patients having ferritin levels < 2,500 ng/ml. A significant positive correlation of increasing serum ferritin levels was observed with ALT (r= 0.682), to a lesser extent with AST (r = 0.532), and only a weak correlation with serum bilirubin (r = 0.350)

**Conclusion:**

Liver damage was caused by increased iron deposition. LFTs should be performed regularly to detect and reduce liver damage by increasing chelation therapy, thereby reducing morbidity and mortality due to thalassemia.

## INTRODUCTION

Hemoglobinopathies are the most frequently encountered mono-gene disorders in humans.^1^ Beta Thalassemia major is one such hemoglobinopathy due to reduced or absent synthesis of beta-globin chain, which causes a decreased production of red blood cells and low haemoglobin content, eventually leading to anaemia.^2^ Hemoglobinopathies were earlier restricted to particular geographical regions, but now a more global distribution is observed owing to immigration and ethnic globalization. Repeated blood transfusions are performed to keep haemoglobin levels within a normal range throughout the life of the individual. Nonetheless, complications are commonly observed due to chronic iron overload following repetitive blood transfusions.^3^

In developed countries, patients of thalassemia have a considerably improved life quality and span due to the institution of modern chelation therapy.^4^ Studies conducted previously have shown that ferritin levels < 2500 ng /mL are associated with few side effects, if any.^5^ Conversely, deficient chelation therapy causes transfusional iron to be deposited in the body leading to organ failure, particularly affecting the heart, liver, and endocrine glands.^6^ Tissue injury occurs due to excess iron deposition causing oxidative stress.^7^ Iron excess in the heart and liver is recognised to be a foremost cause of morbidity and mortality in patients of β-thalassemia major (TM) receiving repeated transfusions.^8^

The largest capacity to store extra iron lies in the liver which is liable to harm due to toxicity caused by iron. In some studies on patients of Thalassemia major receiving chronic current transfusion therapy, serum ferritin was found to be positively correlated with liver iron concentration.^9^ Even though, serum ferritin is the most commonly employed method for an indirect evaluation of iron stores in the body of individuals with β-thalassemia, its utility is restricted by the occurrence of various medical conditions including infection, inflammation, and liver disease which all lead to its elevated levels.^10^

There is insufficient data describing the link of iron overload with hepatic injury in thalassaemic patients. This study was conducted to determine the extent of liver damage in multi transfused thalassemia major patients by determining liver enzymes and to correlate with ferritin levels, in order that rigorous chelation therapy be started to avoid irreparable damage to the liver. This study will provide us with local data, thus allowing further research to reduce burden of thalassemia disease complications.

## METHODS

A cross-sectional study was performed, at Islamabad Medical and Dental College and its affiliated Akbar Niazi Teaching Hospital from November 2021 till August 2022. One hundred and thirty-five subjects were randomly selected from thalassemia major patients coming for routine blood transfusion at Akbar Niazi Teaching Hospital. The selected patients were seven years of age and above. All patients were on chronic transfusion therapy (at least eight transfusions annually). At the time of transfusion, chelation therapy (two injections of Desferal 500 mg/vial), was administered intravenously to all patients. Other than this, all patients were on varying levels of chelation therapy. Presence of acute illness, known cardiac problems whether genetic or acquired, serious renal, hepatic, or endocrinal disorders, other hemoglobinopathies, bone marrow transplantation or medication known to affect liver function tests formed part of the exclusion criteria. Consent; written and informed was acquired from adult patients and guardians of underage patients.

After taking the history of patients, measurement of their height and weight was performed. Preceding blood transfusion, arterial pulse rate and blood pressure were recorded. Blood was drawn by aseptic means and serum was obtained after clotting and centrifugation.

### Serum Ferritin:

Serum was analysed for ferritin by CMIA technology, on an automatic immuno-analyser, ARCHITECT 2000SR (Abbott Laboratories).

### Liver function tests:

Alanine transaminase (ALT), aspartate aminotransferase (AST) and serum bilirubin were determined by ELISA technique using commercially available kits.

### Statistical Analysis:

SPSS version 20 was used for data analysis. For qualitative variables**,** a calculation of frequencies and percentages was performed. For quantitative variables, descriptive statistics were used to calculate mean and standard deviation. ANOVA was used to study the statistical differences among the means of the study groups. This was followed by Post-hoc Tukey’s test to determine which groups were significantly different from each other. A p-value of <0.05 was considered statistically significant. Correlation of LFTs with serum ferritin level was studied by calculation of Pearson’s Correlation Coefficient ‘r’. Pearson’s ‘r’ was measured to assess the strength and direction of a linear relationship between serum ferritin levels and LFTs.

### Ethical approval:

It was obtained from the Institutional Review Board of Islamabad Medical & Dental College vide Approval letter No: 102/IMDC/IREB-2021, dated February 5, 2021.

## RESULTS

Our patients ranged in age from 7- 30 years, and were grouped based on their ferritin levels. Taking serum ferritin level up to 2500 ng/ml as moderate iron overload, and at which few if any side effects are observed, we grouped patients accordingly.^11^ Hence, Group-1 comprised patients with ferritin levels < 2500 ng /ml (mean = 2394.64 ±107.79), Group-2 had ferritin levels between 2500 and 5000 ng/ ml (mean = 4617.61 ± 361.55) and Group-3 with ferritin above 5000 ng/ ml (mean = 8748.51 ± 7.94). In groups 1, 2 and 3 the mean serum bilirubin was found to be 0.79, 0.90, and 1.31mg/dL respectively. Mean AST was 28.61 U/L, 35.74 U/L and 54.47 U/L respectively and ALT levels in the three groups were 40.38 (± 8.91), 48.25 (± 11.44) and 70.91 (±12.13) U/L respectively, (Table-I).

**Table-I:** Mean ± SD of serum bilirubin, AST and ALT in patient groups having different ferritin levels.

Subjects (n=135)	Mean ± SD		

	Total Bilirubin mg/dL	AST U/L	ALT U/L
Group 1 (n=31) Ferritin < 2500 ng/mL	0.79±0.51	28.61±10.29	40.388±.91
Group 2 (n=43) Ferritin 2500-5000 ng/mL	0.90±0.53	35.74±13.18	48.25±11.44
Group 3 (n=61) Ferritin >5000 ng/mL	1.31±0.61	54.47±18.22	70.91±12.13

ANOVA followed by post hoc Tukey’s test established a significant difference in mean bilirubin levels of Group-3 on comparison with Group-1 (p=0.000), however, the difference between Group-1 and Group-2 was not significant (p=0.713). Similarly, AST levels bore a significant difference between Group-1 and Group-3 (p= 0.00) and a nonsignificant difference between Group-1 and Group-2 (p= 0.118), (Table-II).

**Table-II:** ANOVA with Post Hoc Tukey test for analysing difference of means of Serum Bilirubin, AST and ALT between pairs of groups.

Comparison groups	Serum bilirubin	AST	ALT
Gp 1 vs gp 2	p = 0.71	p = 0.11	p = 0.01*
Gp 2 vs gp 3	p = 0.001**	p = 0.00**	p =0.00**
Gp 1 vs gp 3	p = 0.00**	p = 0.00**	p = 0.00**

P value < 0.05 is significant.

**Fig.1:**
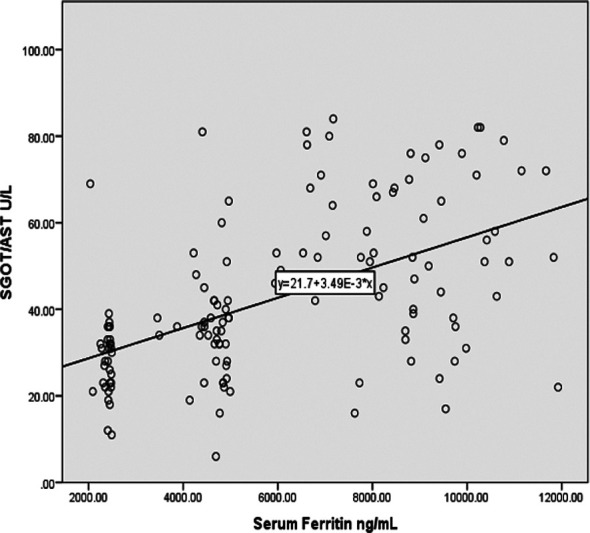
Graph representing a strong positive correlation of AST with ferritin (r= 0.532 **).

**Fig.2:**
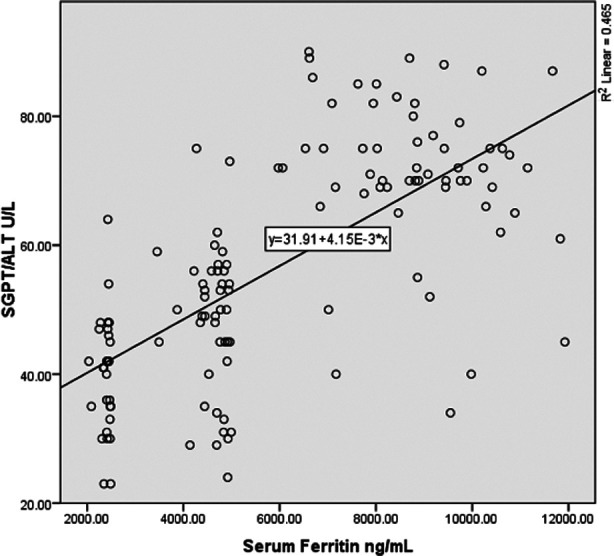
Graph representing a stronger, positive correlation between ALT and ferritin (r= 0.682 **).

However, on comparison of ALT of Group-1 with Group-2 and Group-3, a significant increase was observed in both cases. (p = 0.01, and p = 0.00 respectively) ALT and AST were found to positively correlate with serum ferritin. However, ALT was found to bear a more significant correlation (r = 0.682 **) with serum ferritin as compared to AST (r = 0.532**), (Fig.1 & 2 respectively).

## DISCUSSION

Thalassaemia is an inherited blood disorder in which, depending on the mutation, haemolytic anaemia is observed.^12^ The rapid breakdown of RBCs and repeated blood transfusions lead to increased levels of labile plasma iron. Iron in plasma binds to transferrin, and if the binding capacity of transferrin is overwhelmed, then the excess is stored in various organs e.g.in the liver as ferritin. This accumulation in tissues is likely to cause damage to those organs.^13^ Measurement of serum ferritin provides a feasible and non-invasive method of assessing iron deposition in various tissues as it correlates well with liver iron concentration, a gold standard for determination of iron status.^14^

The liver is amongst the first organs to be damaged by iron overload resulting from repeated blood transfusions and increased intestinal absorption in patients with thalassemia.^15^ Excess iron causes oxidative stress, due to production of Reactive Oxygen Species (ROS) by the Haber Weiss and Fenton reactions. ROS result in lipid peroxidation of cell membranes, mitochondrial impairment, DNA damage leading to cellular dysfunction, apoptosis, fibrosis, and necrosis in target organs, including the myocardium, liver, and endocrine glands.^16^ Hepatocellular damage can be assessed by measuring liver transaminases. Faiq et al. suggested that measurement of serum ALT and AST, be performed regularly to assess liver functioning in thalassemia patients, as an increase in these enzymes is indicative of liver damage.^17^ Moreover, bilirubin, produced by the breakdown of red cells, undergoes hepatic processing and accordingly, its level rises in liver damage.^18^

Our study was conducted to determine the damaging effects of excess iron storage on liver cells. We observed significantly elevated levels of AST, ALT, and serum bilirubin in patients with ferritin levels more than 5000ng/mL. Our results were similar to another study which also reported raised LFTs in transfusion dependent thalassemia patients and confirmed iron deposition to be the root cause.^19^ Banafa et al. in their study also found serum ferritin to be strongly correlated with AST and ALT (*P*<0.05) in thalassemia patients.^20^

A finding of interest in our study was the observation of a more significant correlation of ALT as compared to AST with ferritin levels. This difference may be due to the greater specificity of ALT for hepatic tissues, whereas AST is less specific and present in many tissues.^17^ In another study conducted by Hammod et al. in 2019, on comparison of thalassemia patients with control group, a significant increase in iron and liver enzymes was observed.^21^ Similarly, Suman RL et al in their study also recorded iron deposition to cause compromised functioning of the liver with raised AST and ALT.^22^

In addition to an increase in liver enzymes, a significant fall in serum albumin values and an increase in globulin levels are also attributed to iron overloading of the liver. Moreover, an inverse correlation observed between serum albumin and ALT levels is also indicative of decreased synthetic function of the liver parenchyma, in cases with iron overload.^23^

### Limitations:

Our study would have benefitted from a multi-centre involvement which, however, was not possible due to financial constraints. Moreover, a follow up of patients after giving regular chelation therapy would have added to significantly to our study.

### Significance of the study:

Our study was clinically significant as we observed a derangement of liver function tests in patients with elevated serum ferritin levels, which may be attributed to oxidative stress caused by free labile iron. It is therefore recommended that in our country, for reduction of morbidity and mortality due to thalassemia, liver function be studied at regular intervals. This will enable us to improve management of our patients by instituting an increase in chelation therapy before other organs suffer damage. The result will be an increase in patient longevity, equivalent to that in developed countries.

## CONCLUSION

From our study, we can conclude that patients of β-thalassemia suffered reduced liver function as evidenced by an elevation of serum bilirubin and liver enzymes. Moreover, iron toxicity was found to be more pronounced in patients with serum ferritin levels above 2500 ng/mL.

## Author Contributions:

**AF:** Conceived, designed, and was involved in writing of the manuscript, besides being responsible for integrity of research.

**SS** and **TZ:** Did data collection and manuscript writing.

**IA** and **AI:** Did statistical analysis, review and final approval of manuscript.
